# Case Report: Tuberous sclerosis complex-associated hemihypertrophy successfully treated with mTOR inhibitor sirolimus

**DOI:** 10.3389/fped.2024.1333064

**Published:** 2024-02-21

**Authors:** Konomi Shimoda, Hiroyuki Iwasaki, Yoko Mizuno, Masafumi Seki, Masakazu Mimaki, Motohiro Kato, Aya Shinozaki-Ushiku, Harushi Mori, Seishi Ogawa, Masashi Mizuguchi

**Affiliations:** ^1^ Department of Pediatrics, Graduate School of Medicine, The University of Tokyo, Tokyo, Japan; ^2^ Department of Pediatrics, National Rehabilitation Center for Children with Disabilities, Tokyo, Japan; ^3^ Department of Pathology, Graduate School of Medicine, The University of Tokyo, Tokyo, Japan; ^4^ Department of Radiology, Graduate School of Medicine, The University of Tokyo, Tokyo, Japan; ^5^ Department of Pathology and Tumor Biology, Graduate School of Medicine, Kyoto University, Kyoto, Japan; ^6^ Institute for the Advanced Study of Human Biology, Kyoto University, Kyoto, Japan; ^7^ Department of Developmental Medical Sciences, Graduate School of Medicine, The University of Tokyo, Tokyo, Japan

**Keywords:** tuberous sclerosis complex, hemihypertrophy, limb overgrowth, somatic mutation, loss of heterozygosity, pharmacological treatment, mTOR inhibitor

## Abstract

Tuberous sclerosis complex (TSC) is an autosomal dominant disorder caused by a mutation in either of the two tumor suppressor genes, *TSC1* and *TSC2*. Due to dysregulated activity of the mammalian target of rapamycin (mTOR) pathway, hamartomas or benign tumors frequently occur in many organs and are often treated with mTOR inhibitors. Hemihypertrophy is a rare complication of TSC. Although not being a tumor, progressive overgrowth of the affected limb may cause cosmetic and functional problems, for which the efficacy of mTOR inhibitors has not been reported previously. We herein report a case of TSC-associated hemihypertrophy. In this case, genetic studies revealed *TS*C*1* loss of heterozygosity as the cause of hemihypertrophy. Clinically, pharmacological treatment with an mTOR inhibitor sirolimus successfully ameliorated cosmetic and functional problems with no intolerable adverse effects.

## 1 Introduction

Tuberous sclerosis complex (TSC) is a multisystem disorder caused by a mutation in either the *TSC1* or *TSC2* gene (1). Despite autosomal dominant inheritance, sporadic cases account for about 60% of total cases. In TSC, dysfunction of *TSC1/TSC2* causes dysregulated activation of the phosphatidylinositol-3-kinase (PI3K)/AKT/ mammalian target of rapamycin (mTOR) pathway leading to tumorigenesis (2, 3). TSC-associated benign tumors, or hamartomas, are caused by somatic mutations, typically loss of heterozygosity (LOH) (4), and are treated with mTOR inhibitors, such as sirolimus and everolimus (5, 6).

Hemihypertrophy is a rare complication of TSC (7). Currently, little information is available about its pathogenesis and treatment. Here we report a case of TSC-associated hemihypertrophy. In this case, genetic analysis of the hypertrophic tissue revealed segmental isodisomy of chromosome 9q with *TSC1* LOH and pharmacological treatment with sirolimus was successful.

## 2 Case description

**Figure 1:**
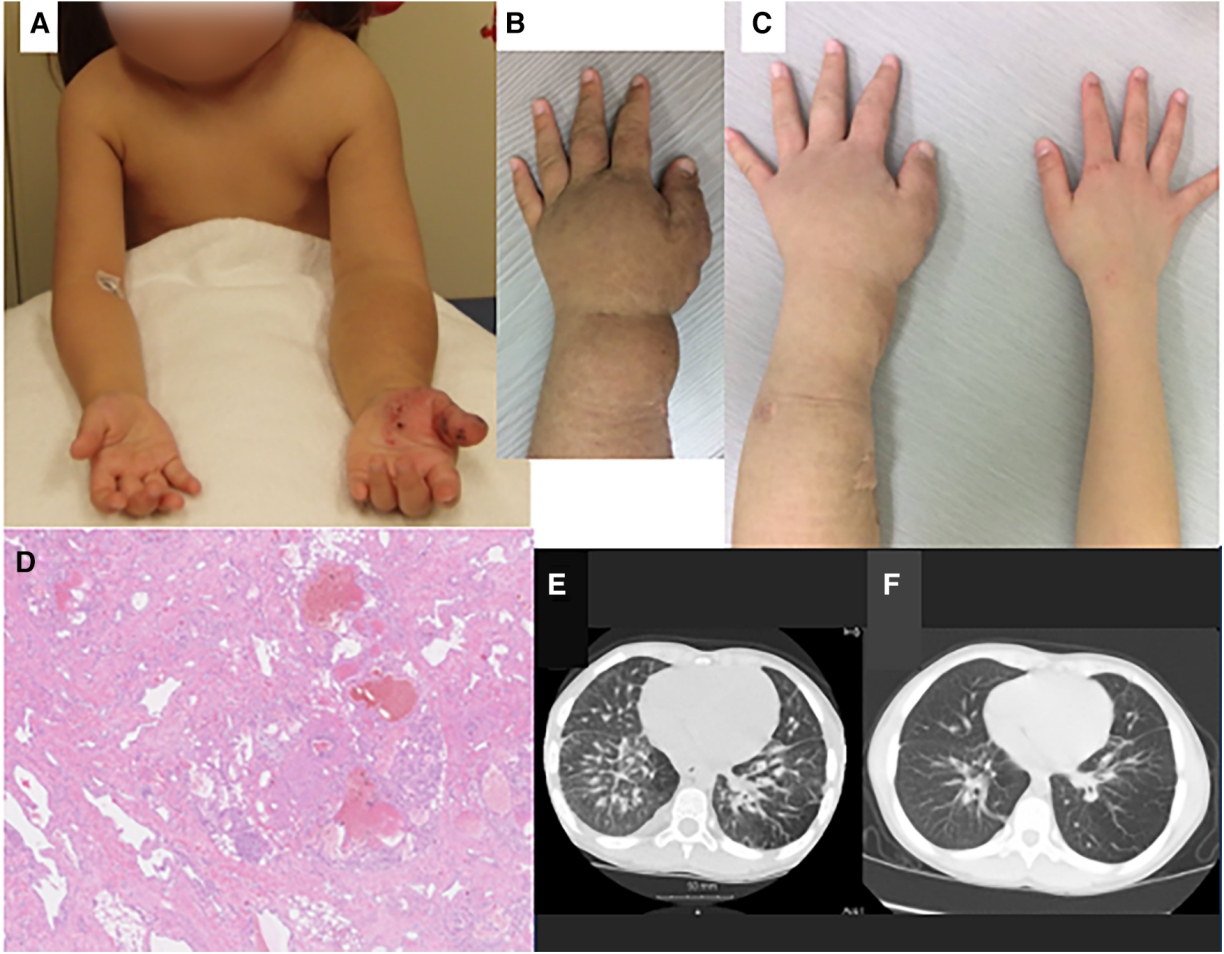
Clinical (**A**–**C**), pathologic (**D**) and radiologic findings (**E**, **F**) of the case. (**A**) At the age of 5 years, there was severe overgrowth of the left arm, with skin pigmentation over the forearm and angiokeratomas over the hand. (**B**) Immediately before treatment with sirolimus (age, 7 years and 7 months), the left forearm showed marked swelling and skin pigmentation. (**C**) After sirolimus treatment (age, 9 years and 1month), swelling and pigmentation improved. (**D**) Histopathology of the resected tissue of left forearm showed vascular malformation: proliferation of lymphatic and small blood vessels, with deformation of arteries and veins and dilatation of lymphatic vessels. (**E**) Chest computed tomography before treatment with sirolimus (age, 7 years and 0 month) showed thickening of the pulmonary interstitium and partial emphysematous change of the lung field. (**F**) After sirolimus treatment (age, 8 years and 9 months), these changes improved. There is in the right lung arteriovenous malformation.

The patient is a Japanese girl born to a family with no history of TSC. In the fetal period, ultrasonography detected multiple hyper-echoic nodules in the heart suggestive of cardiac rhabdomyomas. After 38 weeks of gestation, she was born by Caesarean section, with birthweight of 2,898 g. In infancy, cranial magnetic resonance imaging (MRI) showed multiple subependymal nodules in the brain. She was diagnosed with TSC but had neither epileptic seizures nor developmental delay. Hypertrophy of the left arm, back neck and face appeared soon after birth and progressed thereafter. She also had splenomegaly and thrombocytopenia, with a platelet count of 40–70 × 10^9^/L. By 4 years of age, overgrowth of the arm had caused marked asymmetry, unstable locomotion and limited flexion of the left elbow. Circumferences of the upper arms were 14.5 cm in the right and 19 cm in the left, and those of the forearms 15 cm in the right and 22 cm in the left. The skin on the left hand and forearm showed pigmentation and angiokeratomas (Figure 1A), a skin manifestation rarely noted in TSC (8). The liver and spleen were palpable 1 cm and 6 cm, respectively, below the costal margin. Cranial computed tomography (CT) and MRI visualized hypertrophy of soft tissues in the left back neck, as well as the presence of aneurysms, one in the right vertebral to basilar artery and another in the cavernous portion of left internal carotid artery. There was around the dentate process a nodule constricting the spinal canal at the level of the first cervical spine, which was suspected to be a periodontoid pseudotumor (9). Chest CT and abdominal MRI showed hypertrophy of soft tissues of the left arm, as well as moderate splenomegaly and mild hepatomegaly.

To relieve functional (motor) and cosmetic problems, medication with propranolol was tried (10), but there was no improvement. She then underwent partial resection to reduce the volume of hemihypertrophy, with CO_2_ laser abrasion of angiokeratomas, for three times. The first operation was done for the forearm at the age of 4 years and 9 months, the second for the upper arm at 6 years and 1 month, and the third for the back neck at 7 years and 2 months. Pathological examination of the resected specimen revealed proliferation and deformity of lymphatic and small blood vessels in the subcutaneous tissue. A pathological diagnosis of vascular malformation was made (Figure 1D).

**Figure 2:**
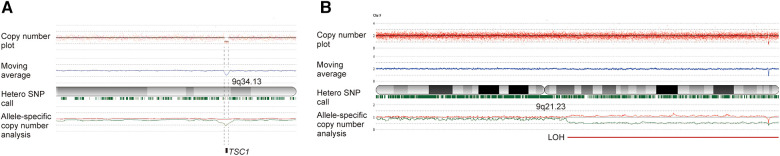
Genetic analyses using SNP array using human reference genome GRCh37/hg19 demonstrated the germline and somatic mutation in this case. (**A**) Analysis of the peripheral blood found a heterozygous microdeletion of 9q34.13 including the *TSC1* gene, with the *TSC1* gene copy number being approximately one. (**B**) Analysis of the resected hypertrophic tissue detected isodisomy of 9q21.23-9qter, resulting in *TSC1* loss of heterozygosity (LOH), with the *TSC1* gene copy number smaller than one. The slight shift in the allele-specific analysis and the retention of marks indicating heterozygosity suggest mosaicism. The red plot displays the total copy number for each probe, along with the moving average indicated by a blue line. Below the cytoband, green marks signify heterozygous SNPs. In allele-specific analysis, the larger and smaller alleles are shown in red and green, respectively.

Genetic studies were conducted using the peripheral blood and resected tissue of the left arm. Single nucleotide polymorphism (SNP) array analyses using the Cytoscan HD array (Affymetrix, Santa Clara, CA) and CNAG/AsCNAR algorithm (11) detected in the blood a deletion of chromosome 9q34.13, from chr9;135664166 to chr9;135851161, including the *TSC1* gene, and in the hypertrophic arm tissue isodisomy of 9q21.23-qter causing allelic loss of the *TSC1* gene (Figure 2). Hemihypertrophy was thus demonstrated to be caused by *TSC1* LOH resulting from the germline and somatic mutations.

After each surgery, her functional and cosmetic problems temporarily ameliorated, but subsequently worsened because of regrowth of the hypertrophic tissues. At 7 years of age, she showed marked overgrowth of the entire left arm, with pigmentation of the skin over the hand and forearm (Figure 1B). Due to asymmetry of the arms, she was poor at keeping balance in motion; she could neither run nor stand on a balance beam. Due to limited flexion of the left elbow, she was unable to touch the left shoulder with the left hand and was clumsy in wearing a shirt. She also had exertional dyspnea; she walked upstairs very slowly, taking a break at every landing. Chest CT showed thickening of the bronchial walls and interlobular septa in the bilateral lungs, partial emphysematous changes in the lung field (Figure 1E), and an arteriovenous fistula in the right lower lobe (S7) of the right lung. The absence of thin-walled cysts excluded the diagnosis of lymphangioleiomyomatosis (LAM), whereas thickening of the interlobular septa was considered to represent lymphedema and/or other abnormalities in the pulmonary lymphatic system similar to those seen in LAM (12). Pulmonary function test revealed a mild impairment of the lungs, with forced expiratory volume 1.0 s % (%FEV_1.0_) of 65.5%.

Chemotherapy with oral administration of sirolimus was started at 7 years and 7 months of age, with a dose of 0.5–1.0 mg/m^2^/day (once a day) and blood sirolimus level of 1.1–1.9 ng/ml. After several months, both cosmetic and functional improvements were noted. Hypertrophy and pigmentation of the left arm ameliorated markedly (Figure 1C). CT and MRI demonstrated reduction in the volume of soft tissues of the left arm and back neck. MRI volumetry confirmed reduction of the left arm hypertrophy and hepatosplenomegaly (Table 1). Chest CT showed improvement of the pulmonary lesions (Figure 1F). She became able to walk on a balance beam, to touch the left shoulder with the ipsilateral hand, and to go upstairs quickly without taking a rest. Platelet count increased to 100 × 10^9^/L, and %FEV_1.0_ to 70.5%. The effects of sirolimus for splenomegaly and thrombocytopenia mimicked those for Kasabach-Merritt phenomenon in kaposiform hemangioendothelioma reported previously (13). Adverse effects of sirolimus were minimal, being limited to mild stomatitis. Currently at the age of 15 years, she is still undergoing maintenance treatment with sirolimus and attending school in good health.

**Table 1:** Volume changes of tissues and organs determined by magnetic resonance volumetry.

Tissue/Organ	Hypertrophy	Before treatment (age, 7 years and 6 months) (mm^3^)	After treatment (age, 9 years and 2 months) (mm^3^)	Volume change
Left dorsum of hand	Yes	70,544	52,296	−26%
Left forearm	Yes	537,464	495,912	−8%
Left upper arm	Yes	565,088	531,808	−6%
Liver	Yes	524,406	462,726	−12%
Spleen	Yes	367,914	267,984	−27%
Left kidney	No	42,240	52,320	+24%
Right kidney	No	43,818	54,906	+25%

## 3 Discussion

We herein reported a rare case of TSC-associated hemihypertrophy. In this case, the site of hypertrophy included the left arm, back neck and face, as well as the spleen and liver. The germline mutation was identified as a microdeletion involving one allele of the *TSC1* gene, and the somatic mutation as isodisomy resulting in *TSC1* LOH. Pharmacological treatment with an mTOR inhibitor, sirolimus, was successful in improving hypertrophy of the tissues and organs, as well as difficulty in walking/wearing, exertional dyspnea and thrombocytopenia, resulting from its effects in the left arm, lungs and spleen, respectively.

TSC is a multisystem disorder characterized clinically by the occurrence of various benign tumors or hamartomas in many organs such as the skin, brain, heart, lungs and kidneys. Except for the heart tumor (cardiac rhabdomyoma), most of these tumors progressively increase in size to cause cosmetic and/or functional problems. Genetically, TSC is caused by a loss-of-function mutation in either of the two genes, *TSC1* and *TSC2*, encoding hamartin and tuberin, respectively, which bind to each other to form a tumor suppressor complex locating at the midstream of PI3K/ AKT/mTOR signaling pathway and negatively regulating its activity (2, 3). The occurrence of TSC-associated hamartomas is explained by two-hit hypothesis. The first hit is a germline mutation in one *TSC1* or *TSC2* allele, whereas the second hit a somatic mutation affecting the other allele: deletion leading to LOH in some tumors and a point mutation in others (4, 14). In the treatment of the TSC-associated hamartomas, such as those in the brain (subependymal giant cell astrocytoma), kidneys (angiomyolipoma, AML), lungs (LAM) and skin (angiofibroma), the mTOR inhibitors, such as sirolimus (rapamycin) and everolimus, have been proven to be safe and efficacious, and are widely used in clinical practice: oral everolimus for SEGA and renal AML (15, 16), oral sirolimus for pulmonary LAM (17) and topical sirolimus for facial angiofibromas (18).

Hemihypertrophy, or unilateral limb overgrowth, is a rare complication of TSC reportedly seen in 3% of TSC patients (7). Our literature review found at least 10 cases of TSC-associated hemihypertrophy affecting one entire limb (19–25) (Table 2). Pathologically, hypertrophic tissues consist of an excess of soft (adipose) and hard (osseous) tissues, hypertrophy and/or malformation of vascular/lymphatic tissues and lymphedema, with the predominant component varying among the cases. Patients with remarkable vascular anomalies and angiomas may meet the diagnostic criteria of Klippel-Trenaunay-Weber (KTW) syndrome (19, 22). Our case showed findings resembling those of KTW syndrome, although she had no angioma in the region of overgrowth.

**Table 2:** Reported cases of TSC-associated hemihypertrophy.

	Reference	Sex	Mutation 1	Mutation 2	Site of overgrowth	Vascular pathology	Bone hypertrophy	Surgical and other treatment	mTOR inhibitor treatment
1	Troost et al. (19)	Female	Unknown	Unknown	Left leg	AVM, KTW	Yes	Not described	No
2	Ortonne et al. (20)	Male	Unknown	Unknown	Left arm	Radial artery agenesis	Yes	Not described	No
3	Reddy et al. (21)	Male	Unknown	Unknown	Left leg (soft tissue)	No	Yes	Not described	Not described
4	Assefa and Alemie (22)	Female	Unknown	Unknown	Left arm	KTW	Yes	None	No
5	Navarre and Poitras (23)	Male	*TSC2*	Unknown	Left leg	Vascular lymphatic malformation	No	Surgery for chronic wounds	No
6	Jenkins et al. (24)	Female	*TSC1*	Unknown	Left arm	Capillary venous and lymphatic malformation, AVM	Yes	Compression, Physiotherapy, Pulsed dye laser	Everolimus with no effect
7		Female	*TSC2*	Unknown	Right leg (subcutaneous adipose tissue)	No	Not stated	Lymphatic massage, Compression	Sirolimus with minimal effect
8		Female	*TSC2*	Unknown	Left leg	No	No	Not described	Not described
9		Female	*TSC2-PKD1*	Unknown	Right arm	Lymphedema	Not stated	Compression, Manual drainage	Sirolimus with no effect
10	Tessarech et al. (25)	Female	*TSC2*	*TSC2* (mosaic point mutation)	Right arm, Liver, Spleen	Portal vein distension	Yes	Not described	Not described
	This study	Female	*TSC1*	*TSC1* (isodisomy causing LOH)	Left arm, Neck, Face, Liver, Spleen	Vascular lymphatic malformation, Pulmonary AVM	No	Partial resection	Sirolimus with significant effect

AVM, arteriovenous malformation; KTW, Klippel-Trenaunay-Weber syndrome; LOH, loss of heterozygosity.

About the pathogenesis of TSC-associated hemihypertrophy, information is very limited. To the best of our knowledge, there is only one previous report demonstrating the two-hit mechanism: a germline mutation of *TSC2* intragenic deletion and a somatic mutation of mosaic *TSC2* missense mutation (25). Our patient is the second case of a proven second-hit mutation, and the first of proven LOH.

Despite pathological difference from tumors, clinical features of hemihypertrophy resemble those of tumors in that the lesions progressively enlarge and eventually cause cosmetic and functional problems, requiring treatment in severe cases. Physical and surgical treatments are conducted in many cases (23, 24). However, the effects of surgery are often temporary due to regrowth of hypertrophic tissues, as was noted also in our case. Oral medication with an mTOR inhibitor, sirolimus or everolimus, has been tried by a previous study for three cases, however, there was only minimal effect in one patient and no improvement in the other two (24) (Table 2). These disappointing outcomes were in sharp contrast with the good response to sirolimus of our case whose hypertrophic tissues/organs showed a clinically significant reduction in volume, whereas non-hypertrophic ones continued normal growth.

Although reasons for the difference between our case and the previous ones are unclear, there are two possible explanations. The first assumes difference in the types of second hit, which was identified as *TSC1* allelic loss in our case but remained unknown in the previous cases (24). The etiology and pathogenesis of hemihypertrophy involve multiple factors. There are multiple causative genes, such as *PIK3CA*, *KRAS*, *PTEN*, *MAP2K3*, *GNAQ*, *TBC1D4* and *TEK* (10). The second hit in TSC-associated hemihypertrophy may possibly affect any of these genes, instead of the *TSC1* and *TSC2* genes. In the molecular situation of trans-heterozygous or “mixed” *TSC1/2* and another gene changes, the efficacy of an mTOR inhibitor is expected to be smaller compared to compound heterozygous or “pure” *TSC1/2* changes. The second explanation concerns differences in the tissue components of hypertrophic limbs. The predominant tissue was small blood/lymphatic vessels in our case, whereas in the previous cases, it was arteriovenous malformation with fast blood flow in one case, subcutaneous adipose tissue in one, and not mentioned in the remaining one (24). Previous studies on the effects of oral mTOR inhibitors for TSC-associated hamartomas have shown that their effects are better in tumors rich in small blood vessels, such as facial angiofibromas and angiomatous/myomatous components of renal AMLs, than in vascular-poor ones, such as ungal fibromas and lipomatous components of AMLs (26, 27). The same explanation may also be tenable in the treatment of hemihypertrophy. In this context, it is noteworthy that oral sirolimus is a therapeutic option in capillary lymphatic venous malformations associated with multiple syndromes (28).

In conclusion, we reported a case of TSC-associated hemihypertrophy caused by LOH of the *TSC1* gene locus. In this case, long-term treatment with oral sirolimus ameliorated cosmetic and functional problems caused by hemihypertrophy, with no intolerable adverse effects. This study is the first report of successful treatment of TSC-associated hemihypertrophy with an mTOR inhibitor. Whether genetic changes, histopathologic features or other factors account for the therapeutic success remains to be elucidated by future studies on additional cases.

## Data Availability

The original contributions presented in the study are included in the article/Supplementary Material, further inquiries can be directed to the corresponding author.
